# Polymorphisms in *Protein Tyrosine Phosphatase Non-receptor Type 2 and 22 (PTPN2/22)* Are Linked to Hyper-Proliferative T-Cells and Susceptibility to *Mycobacteria* in Rheumatoid Arthritis

**DOI:** 10.3389/fcimb.2018.00011

**Published:** 2018-01-25

**Authors:** Robert C. Sharp, Shazia A. Beg, Saleh A. Naser

**Affiliations:** ^1^Burnett School of Biomedical Sciences, University of Central Florida College of Medicine, Orlando, FL, United States; ^2^Health Center, Universtiy of Central Florida College of Medicine, Orlando, FL, United States

**Keywords:** rheumatoid arthritis, *PTPN2*, *PTPN22*, *PTPN2/22*, mycobacteria, SNPs, Crohn's disease

## Abstract

A shared genetic pre-disposition, chronic inflammation, and treatment with similar biologics between Rheumatoid arthritis (RA) and Crohn's disease (CD) have intrigued us to investigate whether the two disorders share trigger association or possible causation. We hypothesized earlier that Single Nucleotide Polymorphisms (SNPs) in the negative regulators *Protein Tyrosine Phosphatase Non-receptor type 2 and 22* (*PTPN2/22)* lead to a dysregulated immune response, susceptibility to environmental triggers, and continued apoptosis as seen in chronic inflammation in RA and CD. To test the hypothesis, peripheral leukocytes samples from 132 consented subjects were genotyped for 9 SNPs in *PTPN2*/22 using TaqMan™ genotyping. The effect of the SNPs on *PTPN2*/22 and *IFN-*γ expression was determined using real time PCR. T-cell proliferation and response to phytohematoagglutonin (PHA) mitogen and mycobacterial antigens were determined by BrdU proliferation assay. Blood samples were also analyzed for the *Mycobacterium avium* subspecies *paratuberculosis* (MAP) *IS900* gene by nPCR. Out of 9 SNPs examined, heterozygous (TC) or minor (CC) alleles of *PTPN2*:*rs478582* occurred in 79% RA compared to 60% healthy controls (*p*-values ≤ 0.05; OR = 2.28). Similarly, heterozygous (GA) or minor (AA) alleles of *PTPN22:rs2476601* occurred in 29% RA compared to 6% healthy controls (*p*-values ≤ 0.05; OR = 5.90). *PTPN2/22* expression in RA was decreased by 1.2-fold compared to healthy controls. *PTPN2:rs478582* upregulated *IFN-*γ in RA by 1.5-fold. Combined *PTPN2:rs478582* and *PTPN22:rs2476601* increased T-cell proliferation by 2.7-fold when treated with PHA. Surprisingly, MAP DNA was detected in 34% of RA samples compared to 8% healthy controls, (*p*-values ≤ 0.05, OR = 5.74). RA samples with *PTPN2:rs478582* and/or *PTPN22:rs2476601* were more positive for MAP than samples without polymorphisms. Combined occurrence of *PTPN2:rs478582* and *PTPN22:rs2476601* in association with the presence of MAP has significantly increased T-cell response and elevated *IFN-*γ expression in RA samples. The data suggest that genetic polymorphisms may play vital role in T-cell regulation, susceptibility to mycobacteria and ultimately response to treatment. This is the first study to report the detection of MAP DNA in the blood of RA patients; further studies are needed using larger number of samples.

## Introduction

**Figure 1:**
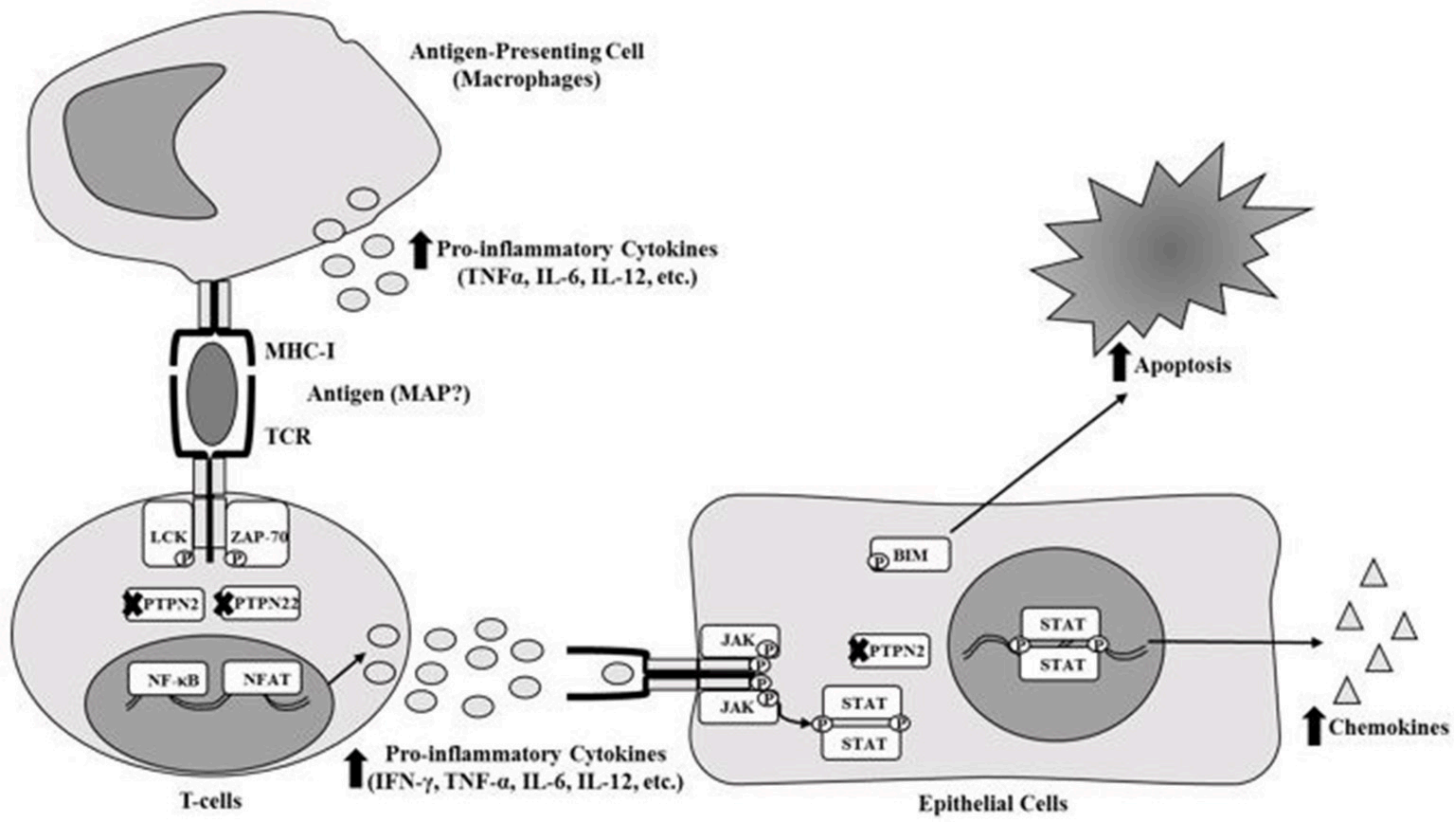
Effect of SNPs in *PTPN2/22* on T-cell response.

Most inflammatory diseases including Rheumatoid Arthritis (RA) have always been classified as autoimmune diseases due to genetic disorders and association with environmental triggers. Genetic predispositions include single nucleotide polymorphisms (SNPs) that affect the alleles of different genes (McInnes and Schett, 2011; Yarwood et al., 2014; Smolen et al., 2016). In RA, several SNPs have been reported in *HLA class 2 histocompatibility antigen, DRB1 beta chain (HLA-DRB1), protein tyrosine phosphatase non-receptor type 22 (PTPN22), cytotoxic T-lymphocyte-associated protein 4 (CTLA4), and cluster of differentiation 40 (CD40)* (McInnes and Schett, 2011; Yarwood et al., 2014; Smolen et al., 2016). SNPs in these genes alter or stimulate the activation and regulation of major components of the immune system (T-cells, B-cells, macrophages, etc.) and osteoclasts which could lead to immune-dysregulation (Brennan and McInnes, 2008; McInnes and Schett, 2011; Smolen et al., 2016). Consequently, this leads to accumulation of immune cells in and around synovial joints and excessive production of anti-CCP, rheumatoid factor autoantibodies (RF) and various pro-inflammatory cytokines such as TNF-α, IFN-γ, IL-1, and IL-6 (Brennan and McInnes, 2008; McInnes and Schett, 2011; Smolen et al., 2016). Specifically, SNPs in immune regulatory genes such as *protein tyrosine phosphatase non-receptor type 2* (*PTPN2*) and *PTPN22* (*PTPN2/22*) could potentially cause these problems in RA. *PTPN2/22* encode proteins that act as phosphatases that negatively regulate the immune response and some regulatory cellular functions (Gurzov et al., 2015; Sharp et al., 2015). Both PTPN2/22 proteins are found in T-cells to remove phosphates from LCK and ZAP-70 of the T-cell receptor (TCR), which in turn stops activation of the TCR (Gurzov et al., 2015; Sharp et al., 2015). PTPN2 protein is also active in epithelial cells around the joints and intestinal tissues, which negatively regulates chemokine production by removing phosphates from STAT1 dimers and reduces apoptosis by removing phosphates from BIM proteins (Gurzov et al., 2015; Sharp et al., 2015). We agree that the prevalence of SNPs in *PTPN2/22* may vary and we support the possibility that the effect on gene expression may be significant which ultimately may void their functions as negative regulators (Figure 1). Consequently, T-cells remain constantly active, leading to hypersecretion of pro-inflammatory cytokines and inflammation along with tissue damage (Gurzov et al., 2015; Sharp et al., 2015).

RA is an idiopathic autoimmune disease with suspected genetic predisposition and environmental triggers association. Due to intense inflammation, hyperplasia of the joints occurs along with cartilage and bone destruction, which leads to extreme pain and deformity of the extremities (Smolen et al., 2007; Aletaha et al., 2010; McInnes and Schett, 2011). RA symptoms include joint swelling and pain of three or more joints, morning stiffness lasting 30 min and subcutaneous rheumatoid nodules (Majithia and Geraci, 2007). Anti-CCP along with RF have also been useful to diagnose RA, more so than erythrocyte sedimentation rate (ESR) and C-reactive protein serum levels (Nishimura et al., 2007; Egerer et al., 2009; Pincus and Sokka, 2009; Aletaha et al., 2010; Taylor et al., 2011). Although, anti-CCP seems to be more specific, but less sensitive than RF in RA diagnosis. Overall, ~30% of patients with RA are negative for anti-CCP (Nishimura et al., 2007; Egerer et al., 2009; Pincus and Sokka, 2009). RF, on the other hand, seems to have lower specificity but higher sensitivity compared to anti-CCP test. Overall, ~30–40% of patients with RA are negative for rheumatoid factor (Nishimura et al., 2007; Egerer et al., 2009; Pincus and Sokka, 2009). The limitation in early and accurate diagnosis of RA affects many patients who are left with continued pain and debilitating suffering. It is imperative that new and improved methods of testing for RA (i.e., genetic testing or identification of potential environmental antigens) is discovered to not only better diagnose RA, but to also find better treatments for the disease as well.

Treatment of inflammatory diseases such as RA and Crohn's disease (CD) includes non-steroid anti-inflammatory drugs (NSAIDs), glucocorticoids, and disease-modifying anti-rheumatic drugs (DMARDs) (Majithia and Geraci, 2007; Smolen et al., 2007, 2016). NSAIDs and glucocorticoids are used for RA patients to help reduce overall pain and stiffness (Majithia and Geraci, 2007; Smolen et al., 2016). However, these medications have a wide-variety of long-term side effects such as ulceration, osteoporosis, hypertension, weight gain, etc., thus NSAIDs and glucocorticoids need to be paired with other medications to reduce the side effects (Majithia and Geraci, 2007; Fakhrudin et al., 2015; Smolen et al., 2016). DMARDs includes synthetic products such as methotrexate, sulfasalazine and hydroxychloroquine and includes biologics such as adalimumab/infliximab (anti-TNF-α), tocilizumab (anti-IL-6 receptor), abatacept (T-cell co-stimulator), and rituximab (B-cell deactivator) (Majithia and Geraci, 2007; Smolen et al., 2016). Using multi-therapy or mono-therapy of different DMARDs is controversial and is continued to be argued among clinicians due to conflicting side effects of each medication. Problems with both synthetic and biological DMARDs continue to be the high risk of developing sides effects including GI intolerance, hypersensitivity to the medication, production of antibodies against the medication, and increasing the risk of developing opportunistic infections such as *Mycobacterium tuberculosis* infection (Bartelds et al., 2007; Dixon et al., 2010; Smolen et al., 2016). DMARDs and synthetic DMARDs are re-classified as DMAIDs when used in inflammatory bowel treatment such as CD (Allen et al., 2017). Infliximab is most commonly prescribed medication for both RA and CD (Kuek et al., 2007; Allen et al., 2017). RA and CD patients share the same treatments, thus it is possible that both RA and CD pathogenesis share common factors involved in disease pathogenesis (Georgiadis et al., 2003; Kuek et al., 2007; Zhernakova et al., 2009; Voight and Cotsapas, 2012; Allen et al., 2017).

Environmental triggers involved in RA include cigarette smoking, air pollutants, and bacteria including *Porphyromonas ginivalis* (*P. gingivalis*) and *Proteus mirablis* (*P. mirablis*) (Klareskog et al., 2011; Fisher et al., 2015; Smolen et al., 2016). Molecular mimicry between a haemolysin protein sequence (ESRRAL) produced by *P. mirablis* and a RA susceptibility sequence (EQRRAA) was reported, thus showing possible connections to genetic pre-disposition and an environmental trigger synergistic threat (Ebringer and Wilson, 2000). Most recently, *Mycobacterium avium* subspecies *paratuberculosis* (MAP) has been associated with other autoimmune diseases including CD, T1D, and possibly in RA (Naser et al., 2009a, 2013; Masala et al., 2011; Sharp et al., 2015). The association of MAP with these inflammatory diseases was based on shared genetic predisposition and molecular mimicry with environmental antigens (Naser et al., 2009a, 2013; Masala et al., 2011; Sharp et al., 2015). MAP infection in a genetically predisposition patient should trigger, exacerbate and possibly dysregulate the immune system by stimulating the production of pro-inflammatory cytokines and, through molecular mimicry, production of autoantibodies (Naser et al., 2009a, 2013; Masala et al., 2011; Sharp et al., 2015). This is the first study designed to explore the effect of an environmental trigger, such as MAP, and SNPs in *PTPN2/22* on gene expression and the consequent effect on T-cells reactivity and inflammation. We hypothesize that SNPs in *PTPN2/22* and, along with MAP infection, causes hyper-proliferative T-cells and overexpression of *IFN-*γ leading to possible inflammation in RA patients.

## Methods

### Clinical samples

Three 4.0-mL K_2_-EDTA coded blood tubes were obtained from 132 consented RA and healthy control subjects that were acquired from the University of Central Florida Health Center. The study was approved by the University of Central Florida Institutional Review Board #IRB00001138. Each subject completed and signed a written consent form before samples were collected. The average age of healthy controls was 30.7 ± 13.4 with a gender ratio of 41.9% male and 58.1% female subjects. The average age of RA patients was 49.9 ± 13.7 with a gender ratio of 11.4% male and 88.6% female subjects. Many factors including the higher prevalence of RA in older females than in males (3:1 ratio) found in other studies, the preference of a female rheumatologists by female RA patients, and the selection of rheumatologists around the area has been noted and considered in this study (Kvien et al., 2006; van Vollenhoven, 2009). Table 1 lists age, gender and other demographic information and current medications for all RA subjects participating in this study. One tube of blood sample was processed for detection of MAP *IS900* DNA. Another tube of blood sample was processed for *PTPN2/22* genotyping and gene expression experiments, whereas the third tube of blood sample was utilized in T-cell proliferation study.

**Table 1:** Demographics, treatment history, and results of MAP and allele frequency of rs478582/rs2476601 in RA samples used in study.

**Sample code**	**Diagnosis**	**Gender**	**Age**	**Medications currently taken**	**MAP status**	PTPN2: rs478582^*^	PTPN22: rs2476601^**^
MAP-1000	RA	F	60	Hydroxychloroquine	−	CC	GG
MAP-1001	RA	M	75	Methotrexate, Prednisone	−	TT	GG
MAP-1003	RA	F	68	Humira®, Methotrexate, Prednisone	−	TC	GG
MAP-1004	RA	F	37	Methotrexate	−	TC	GG
MAP-1002	RA	F	62	Methotrexate	+	TC	GA
MAP-1005	RA	M	30	Methotrexate, Prednisone	−	TC	GG
MAP-1006	RA	F	55	Methotrexate	−	CC	GG
MAP-1007	RA	F	59	Methotrexate, Hydroxychloroquine	−	TC	GG
MAP-1008	RA	F	68	Methotrexate	−	TC	GG
MAP-1009	RA	F	33	Methotrexate, Prednisone	−	TC	GG
MAP-1010	RA	F	62	Methotrexate, Prednisone, Humira®, Sulfasalazine	−	CC	GG
MAP-1011	RA	F	45	Humira®	+	TC	GA
MAP-1012	RA	F	76	Hydroxychloroquine	+	CC	GG
MAP-1013	RA	F	52	Enbrel®, Methotrexate	+	CC	GG
MAP-1014	RA	F	43	Methotrexate	+	TC	GG
MAP-1015	RA	F	47	Enbrel®, Methotrexate	+	TC	GG
MAP-1016	RA	M	48	Methotrexate, Prednisone	+	TC	GG
MAP-1017	RA	F	22	Methotrexate, Simponi®	−	CC	GA
MAP-1019	RA	F	52	Enbrel®	−	TC	GG
MAP-1020	RA	F	60	Orencia®, Methotrexate	−	CC	GA
MAP-1021	RA	F	57	Methotrexate, Simponi®	−	TC	GG
MAP-1023	RA	F	51	Methotrexate, Prednisone	−	CC	GG
MAP-1024	RA	F	62	Methotrexate, Humira®	−	CC	GA
MAP-1022	RA	F	62	Hydroxychloroquine, Methotrexate	−	CC	GG
MAP-1025	RA	F	49	None	−	CC	GG
MAP-1026	RA	F	64	None	−	TC	GG
MAP-1027	RA+IBD	F	56	Prednisone, Xeljanz®	+	CC	GG
MAP-1028	RA+IBD	F	61	Methotrexate, Humira®	−	CC	GA
MAP-1029	RA	F	25	Orencia®, Prednisone	−	CC	GG
MAP-1300	RA	F	39	Orencia®, Methotrexate	−	TC	GA
MAP-1031	RA	F	58	Enbrel®, Leflunomide	+	TT	GG
MAP-1032	RA	F	30	Humira®	−	TC	GG
MAP-1033	RA	F	56	Hydroxychloroquine	+	TC	GG
MAP-1034	RA	F	43	Humira®, Hydroxychloroquine	−	TC	GG
MAP-1035	RA	F	28	Humira®, Methotrexate, Hydroxychloroquine	−	TT	GG
MAP-1036	RA	F	49	Methotrexate, Hydroxychloroquine	+	CC	GA
MAP-1037	RA	F	53	Enbrel®	−	TT	GA
MAP-1039	RA	F	56	Hydroxychloroquine	−	CC	GA
MAP-1040	RA	F	56	Enbrel®, Methotrexate	−	TT	GG
MAP-1041	RA	F	30	Humira®, Prednisone, Leflunomide	−	TC	GG
MAP-1042	RA	F	44	Methotrexate	+	CC	GG
MAP-1043	RA+UC+T1D	F	28	Sulfasalazine, Budesonide	+	TT	GA
MAP-1044	RA	F	39	Hydroxychloroquine	+	TC	GA
MAP-1046	RA	F	54	Hydroxychloroquine	−	CC	GA
MAP-1047	RA	F	65	None	−	TC	GG
MAP-1048	RA	M	65	Methotrexate	+	TT	GA
MAP-1049	RA	F	59	Stelara®	−	TC	GA
MAP-1050	RA	F	73	Humira®	+	TC	GG
MAP-1051	RA	F	34	Prednisone	−	TC	GG
MAP-1052	RA	F	20	Hydroxychloroquine	−	CC	GG
MAP-1053	RA	F	63	Cimzia®, Methotrexate, Predenisone	−	CC	GA
MAP-1054	RA	F	36	Methotrexate	+	TC	GA
MAP-1057	RA	F	51	Methotrexate, Predenisone	−	TC	AA
MAP-1055	RA	F	63	Methotrexate, Hydroxychloroquine, Predenisone	+	TT	GG
MAP-1056	RA	F	47	None	+	TT	GG
MAP-1058	RA	M	42	Methotrexate, Humira®	−	TT	GG
MAP-1059	RA	F	51	Humira®	−	TT	GG
MAP-1060	RA	M	47	Prednisone	+	TC	GG
MAP-1061	RA	F	52	Hydroxychloroquine	+	TC	GG
MAP-1062	RA+T1D	F	50	None	−	TT	GG
MAP-1063	RA+SLE	F	29	Orenseia®, Methotrexate, Predenisone	−	TC	GG
MAP-1064	RA+UC	F	40	None	−	CC	GG
MAP-1065	RA	F	42	Methotrexate, Humira®	−	TT	GA
MAP-1066	RA	F	65	None	−	TC	GG
MAP-1068	RA+CD	F	28	Humira®	−	CC	GG
MAP-1069	RA	M	56	Enbrel®, Methotrexate	−	TC	GG
MAP-1070	RA	F	48	Enbrel®	−	TT	GA
MAP-1067	RA	M	70	Methotrexate, Cimzia®	+	TT	GG
MAP-1071	RA	F	32	Methotrexate	+	TC	GG
MAP-1072	RA	F	58	None	+	TC	GG

RA, Rheumatoid Arthritis; IBD, Inflammatory Bowel Disease; UC, Ulcerative Colitis; T1D, Type 1 Diabetes; SLE, Systemic Lupus Erythematosus; CD, Crohn's Disease;

* TT, Homozygous Major Allele/No SNP; TC, Heterozygous Allele; CC, Homozygous Minor Allele;

** *GG, Homozygous Major Allele/No SNP; GA, Heterozygous Allele; AA, Homozygous Minor Allele*.

### Detection of MAP *IS900* DNA in peripheral leukocytes

Blood sample tubes designated for MAP *IS900* detection were centrifuged at 3,000 RPMs for 10 min at room temperature. A 1.0 mL sample of plasma was transferred to sterile 1.5 mL microcentrifuge tube and was stored at −20°C for further analysis. Buffy coat layer containing peripheral leukocytes were also transferred into new sterile 1.5 mL microcentrifuge tube containing double volume of red cell lysis buffer (ammonium chloride solution, G-Biosciences®). Tubes were then incubated by rocking on a gentle shaker for 10 min, which then were centrifuged at 5,000 RPMs for 5 min at room temperature. The supernatant was removed and purified buffy coat pellets were stored in Tris-EDTA (TE) buffer and subjected to genomic DNA extraction using a modified DNAzol® extraction protocol as follows. Fresh or thawed buffy coat pellets suspended in 1.0 mL DNAzol® reagent was mixed with 400 μL of 100% isopropanol. Tubes were then incubated for 15 min at room temperature followed by centrifugation at 8,000 RPMs for 6 min. The supernatant was discarded and DNA pellets were washed once with 500 μL DNAzol® reagent and centrifuged at 8,000 RPMs for 5 min. Genomic DNA pellets were washed again with 1.0 mL of 75% ethanol and centrifuged at 8,000 RPMs for 5 min. DNA pellets were then dried in a speedvac for 5 min. Dried DNA pellets were dissolved in 20 μL molecular biological grade sterile H_2_O and stored at −20°C for analysis by nPCR. Detection of MAP *IS900* DNA was done following our nPCR protocol and nucleotide primers as described previously (Naser et al., 2004). The presence of a 298 bp band on a 2% agarose gel was indicative of presence of MAP in patient sample. Positive MAP DNA control originated from our UCF4, a culture of clinical strain isolated from CD patient. Negative control tube for each PCR step contained all PCR ingredients except DNA template was used.

### *PTPN2/22* genotyping

Genotyping of *PTPN2/22* for 9 SNPs were performed on DNA from peripheral blood. Genotyping was done at the University of Florida Pharmacotherapy and Translational Research Department (Gainesville, FL) using the TaqMan™ SNP Genotyping Assays (Applied Biosystems™). We investigated 4 SNPs specific to *PTPN2* including *rs1893217, rs2542151, rs7234029*, and *rs478582* along with 5 SNPs specific for *PTPN22* including *rs2476601, rs2488457, rs33996649, rs34209542*, and *rs2476599*. Table 2 summarizes SNPs allele mutations and amino acid mutations used in this study. Briefly, 1 mL blood was stored at −20°C until all samples were collected. DNA extractions were performed on whole blood samples using QIAamp® DNA Blood Mini Kit (Qiagen™) following the manufacturer's protocol. Similarly, TaqMan™ genotyping assays for *PTPN2/22* were performed on DNA samples following manufacturer protocol (Applied Biosystems™). Briefly, reaction mixtures consisted of 2x TaqMan™ Master Mix and 20x Assay Working Stock (primers with VIC and FAM fluorophore attachment) were transferred into a 384-well microtiter plate. DNA samples and controls were then added to the plate which then was subjected to RT-PCR using Applied Biosystems™ QuantStudio™ RT-PCR System. The protocol consisted of 95°C for 10 min for 1 cycle, 92°C for 15 s and 58°C for 1 min for 50 cycles. The plate was read for VIC and FAM fluorophores for each sample at 551 and 517 nm, respectively. Fluorescence of VIC or FAM alone determined that the sample had allele 1 or allele 2, while VIC and FAM together determined that the sample is heterozygous for each allele.

**Table 2:** List of SNPs in *PTPN2/22* examined in this study.

**Gene**	**RS#**	**Mutation**	**Location**	**Mutation phenotype**
*PTPN2*	rs2542151	T>G	5.5 kb Upstream (Espino-Paisan et al., 2011)	High susceptibility to CD, UC, T1D, T2D, RA, and juvenile idiopathic arthritis
	rs1893217	T>C	Intron 7 (Espino-Paisan et al., 2011)	High susceptibility to CD, T1D, MS, RA, and Celiac disease
	rs7234029	A>G	Intronic section (Zhang et al., 2014)	High susceptibility to CD, UC, RA, and juvenile idiopathic arthritis
	rs478582	T>C	Intron 3 (Espino-Paisan et al., 2011)	High susceptibility to T1D, MS, RA, and Celiac disease
*PTPN22*	rs2476601	G>A	R620W (Qu et al., 2005)	High susceptibility to CD, T1D, MS, RA, SLE, and Celiac disease
	rs2488457	C>G	Promoter region (Fan et al., 2015)	High susceptibility to UC, T1D, RA, SLE, and juvenile idiopathic arthritis
	rs33996649	C>T	R263Q (Rodriguez-Rodriguez et al., 2011)	High susceptibility to CD, UC, and RA
	rs34209542	A>G	Intronic section (Skinningsrud et al., 2008)	High susceptibility to T1D, RA, and juvenile idiopathic arthritis
	rs2476599	G>A	Intron 19 (Taniyama et al., 2010)	High susceptibility to RA

### *PTPN2/22* and *IFN-γ* gene expression

A total of 1 mL of fresh whole blood was transferred into 2.0 mL RNA-ase free microcentrifuge tube and was immediately processed for RNA extraction. RNA was isolated from peripheral leukocytes and then used to synthesis cDNA for determining gene expression of *PTPN2/22* and *IFN-*γ via RT-PCR. RNA extraction was performed following the TRIzol® Reagent (Invitrogen) manufacturer's instruction. Briefly,1.0 mL of whole blood was transferred into 2.0 mL RNase free microcentrifuge tubes and centrifuged at 3,000 RPMs for 15 min. Plasma was discarded and buffy coat layer containing peripheral leukocytes were transferred to new RNA-ase free microcentrifuge tubes with double volume of red cell lysis buffer (ammonium chloride solution, G-Biosciences®). Tubes were incubated by rocking on gentle shaker for 10 min which then was centrifuged at 5,000 RPMs for 5 min at room temperature. Supernatant was then removed and peripheral leukocyte pellets were suspended in 1.0 mL of TRIzol®. Tubes were then incubated by rocking on a gentle shaker for 15 min. A volume of 0.2 mL of chloroform was then added to each tube. The mixture was then incubated at room temperature for 3 min. Tubes were then centrifuged at 11,400 RPMs for 15 min at 4°C. The colorless, upper aqueous phase containing RNA was transferred into new 2.0 mL RNA-ase free microcentrifuge tubes. A volume of 0.5 mL of 100% isopropanol was added followed by incubation at room temperature for 10 min. Tubes were then centrifuged at 11,400 RPMs for 10 min at 4°C. RNA pellets were washed in 1 mL of 75% ethanol and then centrifuged at 8,700 RPMs for 5 min at 4°C. RNA pellets were air-dried for 15–30 min and then suspended in 20 μL of RNase free H_2_O and heated at 60°C for 10 min.

cDNA synthesis was performed following the iScript™ Reverse Transcription (Bio-Rad®) manufacturer's instruction. Briefly, 600 ng of RNA from each sample was added to PCR reaction tubes containing 0.2 mL PCR reaction, 4 μL of iScript™ Reverse Transcription (Bio-Rad®), and up to 20 uL RNase free H_2_O. Tubes were then placed in a thermal cycler (MyGene™ Series Pelteir Thermal Cycler) and ran for 5 min at 25°C, 20 min at 46°C, and 1 min at 95°C. Final concentration of cDNA for each sample was 30 ng/μL.

RT-PCR reactions in a 96-well microamp plate consisted of 1 μL of cDNA (30 ng), 10 μL of Fast SYBR Green Mastermix (Thermofisher Scientific®), 1 μL of either *PTPN2, PTPN22*, or *IFN-*γ PrimePCR SYBER Green Assay mix (Bio-Rad®). and 8 μL of molecular biological grade sterile H_2_O. Oligonucleotide primers for 18s RNA gene (forward primer: 5′-GTA ACC CGT TGA ACC CCA TT-3′; reverse primer: 5′-CCA TCC AAT CGG TAG TAG CG-3′) were used as a control and to obtain baseline CT readings. RT-PCR reaction was performed using the 7500 Fast Real-Time PCR System (Applied Biosystems®). Relative mRNA expression levels were calculated using ΔCT (Sample RT-PCR CT reading–18s CT baseline) and using the equation (2^−Δ*CT*^ × 1,000).

### Isolation of peripheral lymphocytes and proliferation assay

Isolation of peripheral lymphocytes was performed using Lymphoprep™ reagent (Axis-Shield®) as described previously (Naser et al., 2009b). A stock of 2X freezing media containing 10.0 mL of 25% human serum albumin (Gemini®), 10.0 mL of sterile RPMI-1640 (Sigma-Aldrich®), and 5.0 mL DMSO was made for the use of preserving lymphocytes for storage at −80°C. Isolated lymphocytes were transferred into 1.0 mL cryogenic vials (Nalgene®) with double the amount of 2x freezing media added to samples and stored at −80°C for future use. Lymphocytes were thawed and washed with cRPMI, which contained 10% sterile heat-inactivated FBS (Sigma-Aldrich®) and 1% sterile antibiotic/antimycotic solution (Sigma-Aldrich®) added to RPMI-1640 before T-cell isolation. T-cell isolation from lymphocyte samples were done using EasySep™ Human T-cell Isolation Kit (Stemcell™) following manufacturer's instruction. Briefly, isolated lymphocytes were transferred into a 2.0 mL round-bottom microcentrifuge tubes. The Isolation Cocktail mixture was added at 50 μL/mL to sample tubes and was incubated at room temperature for 5 min. The RapidSpheres™ mixture was added to the tubes at 40 μL/mL and were placed in the EasySep™ magnet for 3 min. Isolated T-cells were poured from the tubes in the magnet to new 2.0 mL microcentrifuge tubes. T-cells were then counted using trypan blue solution (0.4%, Sigma®) cell viability assay.

T-cell proliferation assay was done using bromodeoxyuridine (BrdU) labeling proliferation ELISA kit (Roche Molecular Biochemicals®) as described previously (Naser et al., 2009b). Phytohematoagglutunin (PHA) was used to evaluate T-cell response. Purified Protein Derivative-like (PPD-like) from MAP was prepared by purification of supernatant from sonicates of protein extract obtained from clinical strain UCF4 culture pellet. It was used to determine T-cell response and prior exposure to MAP antigens. Briefly, 1 × 10^5^ of isolated T-cells were transferred in triplicates onto a 96-well culture plate and were incubated in either RPMI only, PHA (10 μg/mL, Sigma-Aldrich®) or PPD-like (5 μg/mL) along with respected patients' plasma for 72 h at 37°C and 5% CO_2_. T-cells were then labeled with BrdU and incubated for 24 h at 37°C and 5% CO_2_. Cell proliferation was measured through Roche BrdU proliferation ELISA kit as described previously (Naser et al., 2009b). Relative T-cell proliferation levels of samples were compared to blanks (RPMI only) and controls (isolated T-cells in RPMI only) by examining fold change in absorbance reading of each well at 450 nm.

### Statistical analysis

Samples were analyzed for significance using unpaired, two-tailed *t*-tests; unpaired, two-tailed z-score; and odds ratio. GraphPad Prism 7 was used for statistical analysis and creation of graphs. *P*-values ≤ 0.05 were considered significant. Relative mRNA gene expression was determined by the use of ΔCT of the gene of interest found in each sample and the equation 2^−Δ*CT*^ × 1,000 (Sullivan et al., 2013).

## Results

### *Mycobacterium avium* subspecies *Paratuberculosis-IS900* DNA detected in RA

**Figure 2:**
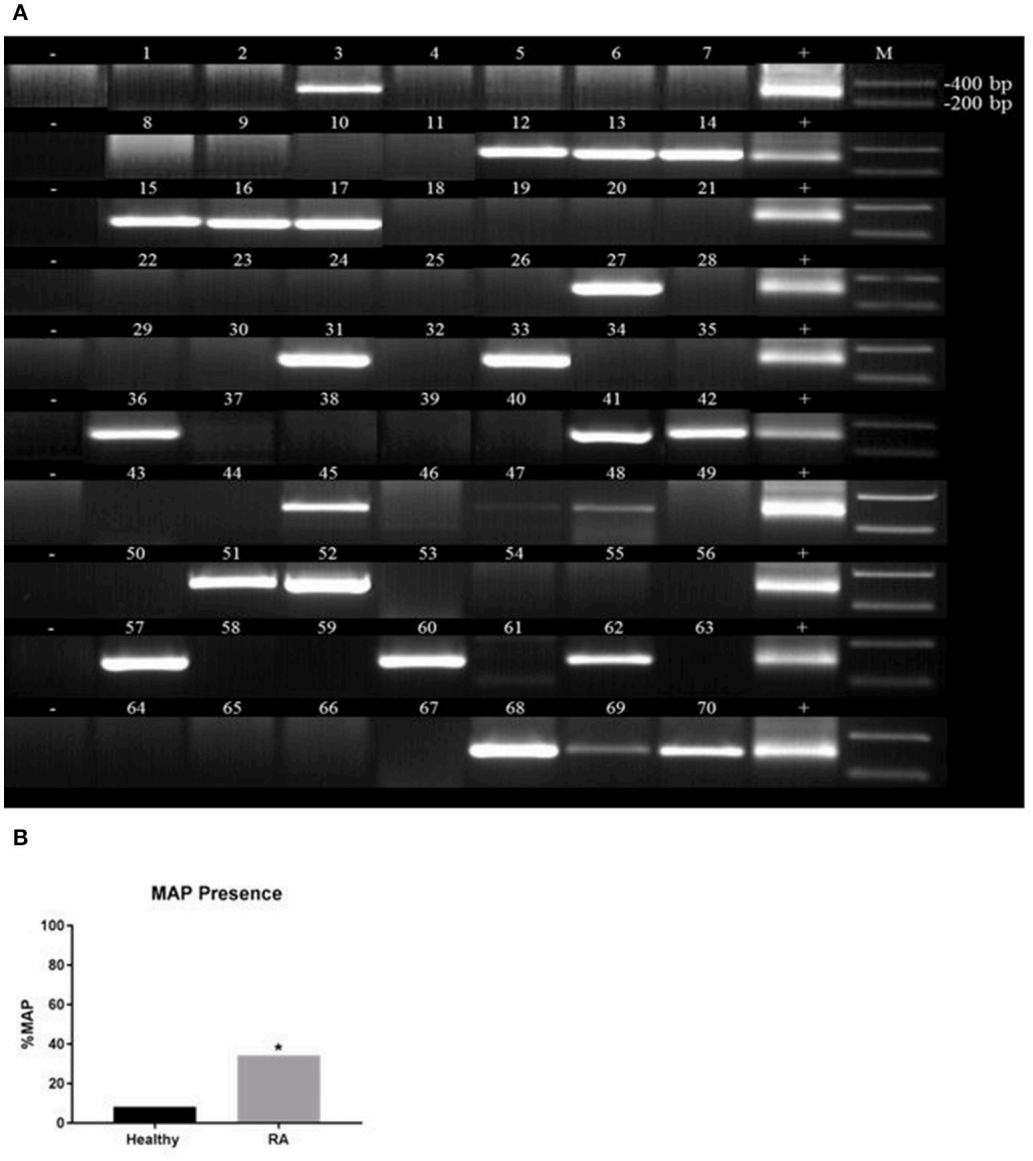
Detection of *Mycobacterium avium subspecies paratuberculosis* (MAP) in blood samples from RA. Nested PCR was performed on genomic DNA from blood samples from RA subjects **(A)** and healthy controls **(B)**. DNA from MAP strain UCF4 was used as a positive control (+); a negative control (without DNA template) was also included (−). M corresponds with molecular weight marker. ^*^P-values ≤ 0.05.

Purified DNA from peripheral leukocytes of 118 subjects (70 RA and 48 healthy controls) was analyzed by nPCR using oligonucleotide primers specific to MAP *IS900*. MAP DNA was detected in blood samples from RA subject as illustrated in Figure 2A. The 298 bp PCR product purified from representative gels was sequenced and BLAST analysis confirmed the identity of MAP, which has previously been used to confirm if patient samples are considered having the MAP infection (Naser et al., 2004, 2009a, 2013). As shown in Figure 2B, out of 70 blood samples from RA subjects, 24 (34.3%) were positive for *MAPbacteremia* compared to only 4 out 48 (8.3%) healthy controls (*p*-value ≤ 0.05). The odds ratio (OR) value was determined to be 5.74 (95% CI: 1.84–17.9; *p*-value ≤ 0.05), where the presence of MAP DNA is most likely to occur in RA patients. MAP bacteria has been successfully re-cultured from at least one RA buffy coat sample (MAP-1015, see Table 1) via BD Bactec™ MGIT™ Para-TB medium (Becton, Dickinson and Company). The cultured sample was confirmed to be MAP positive by way of nPCR as previously mentioned. Culturing of MAP bacteria from other RA patient samples is still ongoing.

### Frequency of SNP alleles in *PTPN2/22* in RA

**Figure 3:**
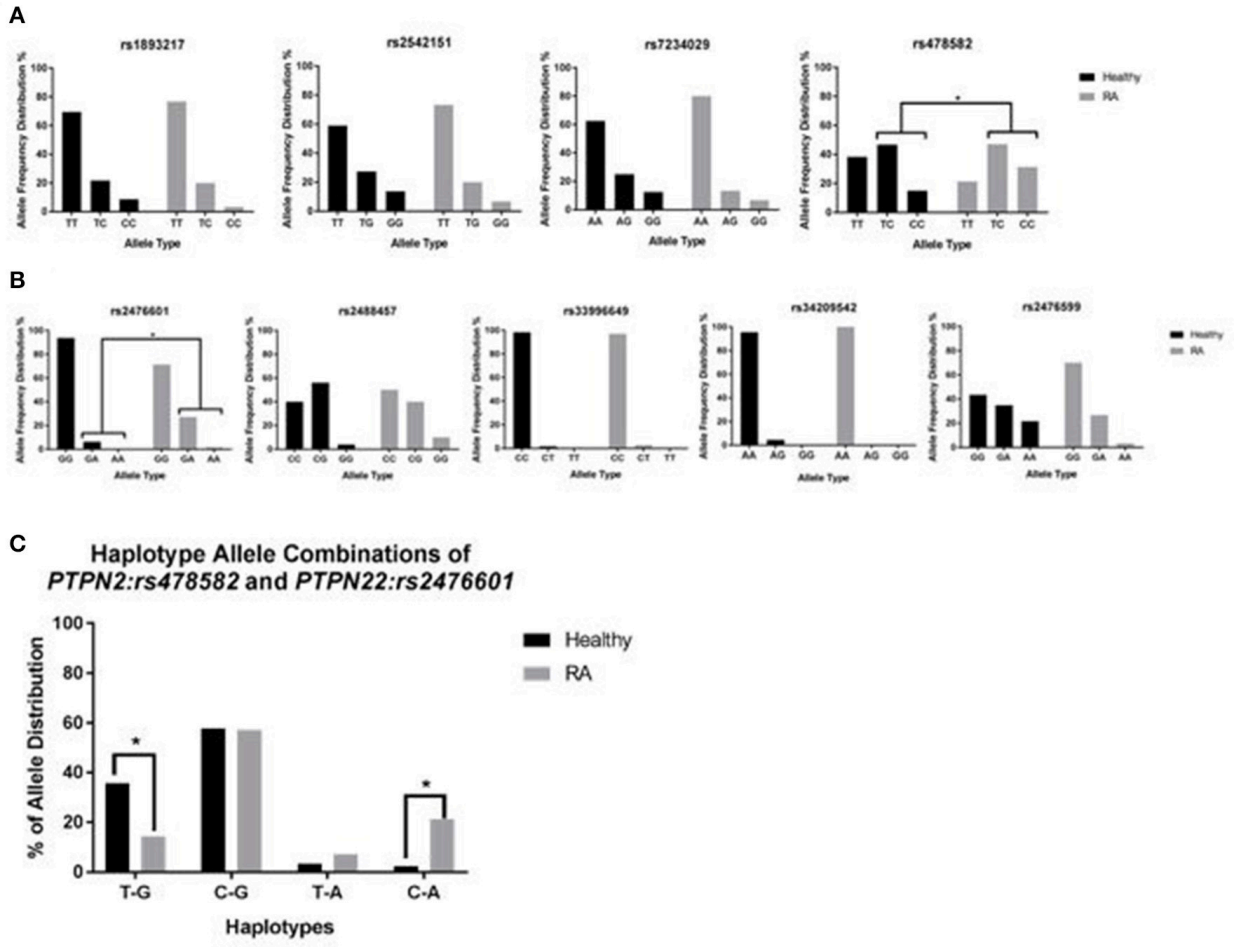
Genotyping of 9 SNPs in *PTPN2/22* in RA. **(A)** Represents the allele frequency in *PTPN2: rs1893217, rs2542151, rs7234029, rs478582*. **(B)** Represents the allele frequency in *PTPN22: rs2476601, rs2488457, rs33996649, rs34209542, rs2476599*. **(C)** Represents haplotype combinations between *PTPN2:rs478582* and *PTPN22:rs2476601* including T-G (major/major), C-G (heterozygous or minor/major), T-A (major/heterozygous or minor), and C-A (minor/minor). ^*^P-values ≤ 0.05.

TaqMan™ genotyping was done on purified DNA from 132 subjects (70 RA and 62 healthy controls). DNA from each subject was analyzed for 4 SNPs specific to *PTPN2* (*rs1893217, rs2542151, rs7234029, rs478582*) and 5 SNPs specific to *PTPN22* (*rs2476601, rs2488457, rs33996649, rs34209542, rs2476599*). Data referred to as homozygous major allele is considered normal/no SNP, while heterozygous allele and homozygous minor allele were considered abnormal and designated as SNP positive. As shown in Figure 3, Out of 4 SNPs specific to *PTPN2, rs478582* was significant in RA since heterozygous (TC) or minor (CC) alleles were detected in 55/70 (78.6%) RA samples compared to 36/60 (60.0%) healthy controls (*p*-value ≤ 0.05, Figure 3A). Specifically, 22/70 (31.4%) minor (CC) alleles were detected in RA compared to 9/60 (15.0%) healthy controls (*p*-value ≤ 0.05), whereas heterozygous (TC) alleles were detected in 33/70 (47.1%) RA compared to 28/60 (46.7%) healthy controls. Out of 5 SNPs specific to *PTPN22, rs2476601* was significant in RA since heterozygous (GA) or minor (AA) alleles were detected in 20/70 (28.6%) RA samples compared to only 4/62 (6.45%) healthy controls (*p*-values ≤ 0.05, Figure 3B). Specifically, heterozygous alleles (GA) were detected in 19/70 (27.1%) RA compared to 4/62 (6.45%) healthy controls (*p*-value ≤ 0.05). There was rare minor (AA) alleles detected in all samples. The OR value for the significance of *PTPN2:rs478582* was 2.28 (95% CI: 1.05–4.93; *p*-value ≤ 0.05) whereas OR value for *PTPN22:rs2476601* was 5.90 (95% CI: 1.89–18.4; *p*-value ≤ 0.05).

For determination of haplotype combinations, we examined the significant SNPs *PTPN2:rs478582* and *PTPN22:rs2476601* allele combinations to confirm the allele distribution among samples (Figure 3C). Examination of the following haplotype combinations were determined in the samples, where *PTPN2:rs478582* and *PTPN22:rs2476601* allele types were combined respectively: T-G, C-G, T-A, and C-A. T-G haplotype (major/major) was found more significantly in healthy controls (21/59 = 35.6%) than in RA samples (10/70 = 14.3%, *p*-value ≤ 0.05). C-G haplotype (heterozygous or minor/major) was found in 40/70 (57.1%) RA samples compared to healthy 34/59 (57.6%). T-A haplotype (major/heterozygous or minor) was found more in RA samples (5/70 = 7.14%), compared to healthy controls (2/59 = 3.39%), while C-A (heterozygous or minor/heterozygous or minor) was found significantly more in RA samples (15/70 = 21.4%) than in healthy controls (2/59 = 2.39%, *p*-value ≤ 0.05). When examining the haplotypes in more detail, CC-GA haplotype was found more significantly (*p*-value ≤ 0.05) in RA patients (8/70 = 11.4%) than in healthy controls (1/59 = 1.69%).

### Effect of *PTPN2:rs478582* and *PTPN22:rs2476601* on *PTPN2/22* expression

Gene expression of *PTPN2/22* in 37 RA and 31 healthy controls were reported. The overall relative mRNA expression of *PTPN2* was lower in RA compared to healthy controls (8.22 ± 5.33 and 10.3 ± 6.95, respectively). Similarly, relative mRNA expression of *PTPN22* was also lower in RA compared to healthy controls (2.55 ± 1.74, and 3.24 ± 1.84, respectively). Examination of relative mRNA expression of *PTPN2/22* in relationship with samples with either *PTPN2:rs478582* or *PTPN22:rs2476601* was examined as seen in Table 3.

**Table 3:** Effect of *PTPN2:rs478582* and *PTPN22:rs2476601* on *PTPN2/22* expression.

**Diagnosis**	***PTPN2*** **expression of samples with** ***PTPN2:rs478582*** **(2**^**−ΔCT**^ **× 1000)**				***PTPN22*** **expression of samples with** ***PTPN22:rs2476601*** **(2**^**−ΔCT**^ **× 1000)**			
	**TT**	**TC**	**CC**	**TC + CC**	**GG**	**GA**	**AA**	**GA + AA**
RA	7.38 ± 4.91 (*N* = 13)	7.42 ± 4.01 (*N* = 15)	10.7 ± 7.33 (*N* = 9)	8.67 ± 5.59 (*N* = 24)	2.41 ± 1.98 (*N* = 24)	2.77 ± 1.28 (*N* = 12)	3.16 *(N* = 1)	2.79 ± 1.23 (*N* = 13)
Healthy	9.49 ± 5.13 (*N* = 8)	10.3 ± 7.01 (*N* = 18)	11.9 ± 9.73 (*N* = 5)	10.6 ± 7.47 (*N* = 23)	3.24 ± 1.91 (*N* = 27)	3.40 ± 1.19 (*N* = 4)	NA	3.40 ± 1.19 (*N* = 4)

The effect of *PTPN2:rs478582* on gene expression was evaluated. The average relative mRNA expression in RA with heterozygous (TC) or minor (CC) allele was 8.67 ± 5.59 (*N* = 24) compared to 10.6 ± 7.47 (*N* = 23) in healthy controls with similar SNPs and lower than healthy controls without SNPs (TT) (9.49 ± 5.13; *N* = 8). Specifically, the average relative mRNA expression in RA with heterozygous (TC) allele was 7.42 ± 4.01; *N* = 15) which is much lower than healthy control with the heterozygous (TC) allele (10.3 ± 7.01; *N* = 18) and normal (TT) healthy controls (9.49 ± 5.13; *N* = 8). The effect of minor (CC) allele on *PTPN2* expression in RA was 10.7 ± 7.33 (*N* = 9) and lower than healthy controls with minor (CC) allele (11.9 ± 9.73; *N* = 5). The effect of *PTPN2:rs478582* on mRNA expression in each subject group was not significant. Among healthy controls, the average relative mRNA expression in samples with heterozygous (TC) or minor (CC) allele was 10.6 ± 7.47 (*N* = 23) compared to 9.49 ± 5.13 (*N* = 8) in normal (TT) samples. The average relative mRNA expression with only heterozygous (TC) allele was 10.3 ± 7.01(*N* = 18), whereas samples with minor (CC) allele had 11.9 ± 9.73 (*N* = 5) compared to normal (TT) healthy controls (9.49 ± 5.13 *N* = 8). Among RA samples, the average relative mRNA expression in samples with the heterozygous (TC) or minor (CC) allele was 8.67 ± 5.59 (*N* = 24) compared to 7.38 ± 4.91 (*N* = 13) in normal (TT) samples. The average relative mRNA expression samples with only heterozygous (TC) allele was 7.42 ± 4.0 (*N* = 15), whereas samples with the minor (CC) allele had 10.7 ± 7.33 (*N* = 9) compared to RA normal (TT) samples (7.38 ± 4.91; *N* = 13). The overall average relative mRNA expression in all samples with heterozygous (TC) or minor allele (CC) was 9.63 ± 6.58 (*N* = 47) compared to 8.19 ± 4.98 (*N* = 21) in samples without any SNP. Specifically, the average relative mRNA expression in all samples with only heterozygous (TC) allele was 8.99 ± 5.94 (*N* = 33) and with only the minor (CC) allele was 11.2 ± 7.91 (*N* = 14) compared to the samples without any SNP (8.19 ± 4.98; *N* = 21).

Correlation analyses were also performed to determine if *PTPN22:rs2476601* alters *PTPN22* expression. The average relative mRNA expression in RA with heterozygous (GA) or minor allele (AA) was 2.79 ± 1.23 (*N* = 13) compared to 3.40 ± 1.19 (*N* = 4) in healthy controls with similar SNP and normal (GG) healthy control (3.24 ± 1.91, *N* = 27). Specifically, the average relative mRNA expression in RA with only heterozygous (GA) allele was 2.77 ± 1.28 (*N* = 12) compared to 3.40 ± 1.19 (*N* = 4) in healthy controls with similar SNP and normal (GG) healthy controls (3.24 ± 1.91; *N* = 27). There was rare occurrence of minor (AA) allele in all samples. Among each group, there was not any significance. Among healthy controls, the average relative mRNA expression in samples with heterozygous (GA) or minor (AA) allele was 3.40 ± 1.19 (*N* = 4) compared to 3.24 ± 1.91 (*N* = 27) normal (GG) samples. The average relative mRNA expression with only heterozygous (GA) allele was 3.40 ± 1.19 (*N* = 4), where there were no samples with minor (AA) allele. Among RA samples, the average relative mRNA expression in samples with the heterozygous (GA) or minor (AA) allele was 2.79 ± 1.23 (*N* = 13) compared to 2.41 ± 1.98 (*N* = 24) in normal (GG) samples. The average relative mRNA expression samples with heterozygous (GA) allele only was 2.77 ± 1.28 (*N* = 12) compared to 3.16 in minor (AA) allele. There was no significant difference in the overall average relative mRNA expression in all samples with heterozygous (GA) or minor allele (AA) and samples without any SNP. Specifically, the average relative mRNA expression in all samples with only heterozygous (GA) allele was 2.92 ± 1.25 (*N* = 16) and with only the minor (AA) allele was 3.16 (*N* = 1) compared to the samples without any SNP (2.85 ± 1.97, *N* = 51).

### Effect of *PTPN2:rs478582* and *PTPN22:rs2476601* on T-cell response

**Figure 4:**
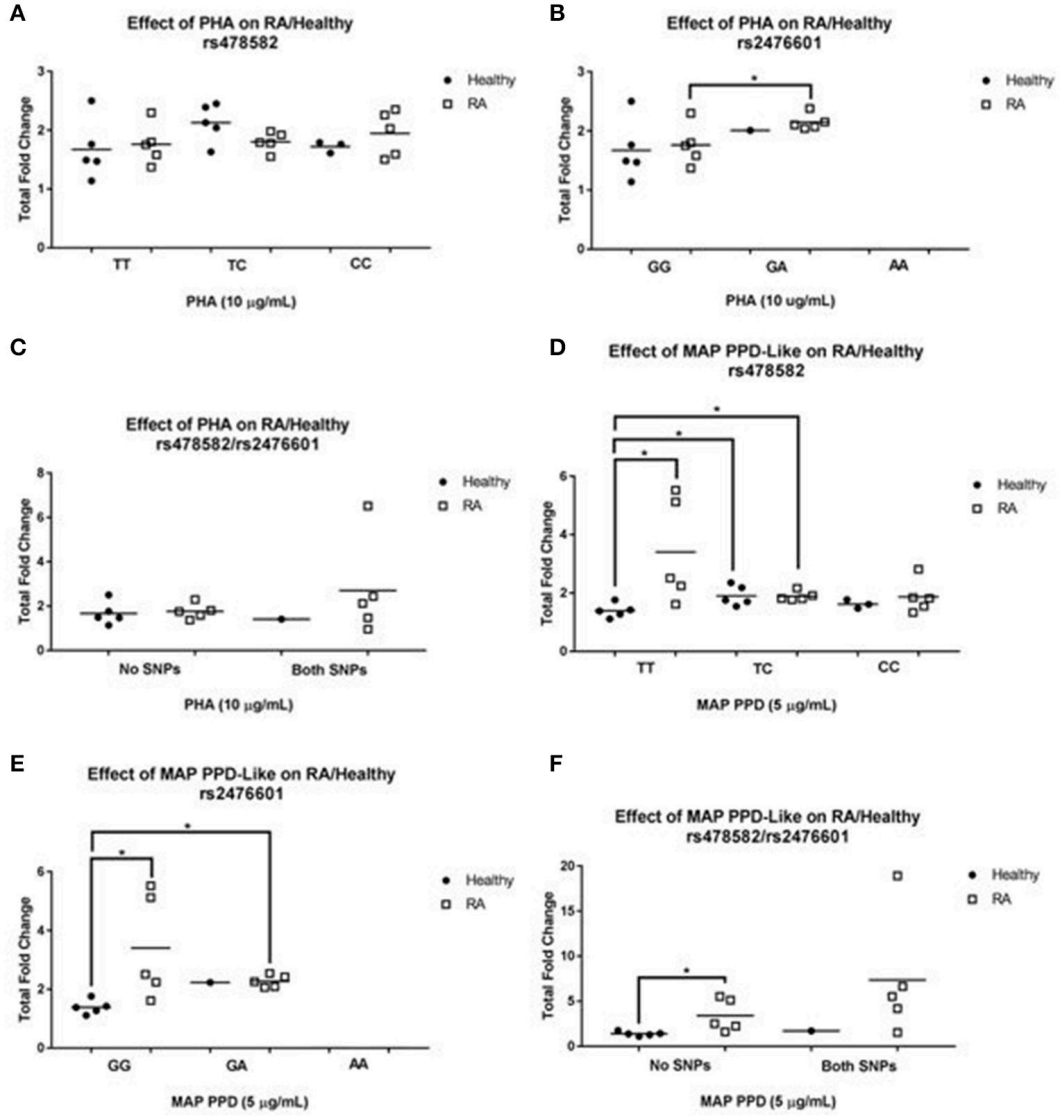
T-cell Response in RA Associated with *PTPN2:rs478582* and *PTPN22:rs2476601*. **(A,B)** Against Phytohematoagglutonin (PHA). **(D,E)** Against MAP Purified Protein Derivative-Like (PPD-Like). *PTPN2:rs478582-* heterozygous allele (TC), minor allele (CC), and wild type (TT). *PTPN22:rs2476601*-heterozygous allele (GA), minor allele (AA), and wild type (GG). The effect of combined SNPs in *PTPN2/22* in T-cells induced with either PHA or MAP PPD-like is illustrated in **(C,F)**. ^*^P-values ≤ 0.05.

To evaluate the effect of *PTPN2:rs478582* and/or *PTPN22:rs2476601* on T-cell function, we treated purified T-cells from RA (*N* = 25) and healthy controls (*N* = 15) with PHA and MAP PPD-like and measured T-cell proliferative response (Figure 4). T-cells were isolated and purified from clinical samples, which tested positive for heterozygous and/or homozygous minor alleles for *PTPN2:rs478582* and/or *PTPN22:rs2476601*.

#### Effect of *PTPN2:rs478582* on T-cell response

Unlike T-cells from RA subjects, there was an increase in T-cell proliferation response between healthy controls with heterozygous (TC) allele (2.1± 0.3-fold increase, *N* = 5) and those without SNP (TT) (1.7 ± 0.5-fold increase, *N* = 5) when induced with PHA (Figure 4A). On the contrary, there was 2.0 ± 0.4-fold increase (*N* = 5) in T-cells response in RA samples with minor (CC) allele compared to a 1.8 ± 0.3-fold increase (*N* = 5) in RA normal (TT) T-cells. There was no difference in T-cell response in healthy controls with (*N* = 3) and without (*N* = 5) minor allele. The effect of heterozygous (TC) allele on T-cell proliferation response from healthy controls treated with MAP PPD-like (Figure 4D) resulted in a 1.9 ± 0.3-fold increase (*N* = 5) compared to only 1.4 ± 0.2-fold increase (*N* = 5) in normal (TT) T-cells from healthy control (*p*-value ≤ 0.05). T-cells from healthy controls with minor (CC) allele responded to MAP PPD-like with 1.6 ± 0.2-fold increase (*N* = 3) compared to 1.4 ± 0.2 (*N* = 5) in normal T-cells from healthy controls. RA samples with heterozygous (TC) allele had a significantly higher T-cell proliferation response fold increase to MAP PPD-like (1.9 ± 0.2, *N* = 5) compared to healthy controls with normal (TT) (1.4 ± 0.2, *N* = 5, *p*-value ≤ 0.05).

#### Effect of *PTPN22:rs2476601* on T-cell response

The effect of heterozygous (GA) allele on T-cell proliferation response from healthy controls treated with PHA (Figure 4B) resulted in a 2.0-fold increase (*N* = 1) compared to only 1.7 ± 0.5-fold increase (*N* = 5) in normal (GG) T-cells from healthy controls. Similarly, T-cells from RA subjects with heterozygous (GA) allele responded with a 2.2 ± 0.1 (*N* = 5) fold increase compared to a 1.8 ± 0.3-fold increase (*N* = 5) in normal (GG) T-cells from RA subjects (*p*-value ≤ 0.05). There were no patient samples with just the minor (AA) allele to do T-cell proliferation. The effect of heterozygous (GA) allele on T-cell proliferation response from healthy controls treated with MAP PPD-like (Figure 4E) resulted in a 2.2 (*N* = 1) fold increase compared to only 1.4 ± 0.2 (*N* = 5) fold increase in normal (GG) T-cells from healthy controls. T-cells from RA samples with heterozygous (GA) allele responded lower (2.3 ± 0.2-fold increase, *N* = 5) to MAP PPD-like than normal T-cells (3.4 ± 1.8-fold increase, *N* = 5). RA samples with normal (GG) T-cells had a significantly higher response to MAP PPD-like (3.4 ± 1.8, *N* = 5) compared to healthy controls with normal (GG) (1.4 ± 0.2, *N* = 5, *p*-value ≤ 0.05). RA samples with heterozygous (GA) allele had a significantly higher T-cell proliferation response to MAP PPD-like (2.3 ± 0.2, *N* = 5) compared to healthy controls with normal (GG) (1.4 ± 0.2, *N* = 5, *p*-value ≤ 0.05).

#### Effect of combined *PTPN2:rs478582* and *PTPN22:rs2476601* on T-cell response

Response of T-cells from RA samples with both SNPs treated with PHA was 2.7 ± 2.2-fold increase (*N* = 5) compared to a 1.8 ± 0.3-fold increase (*N* = 5) in T-cells from RA samples without SNP (Figure 4C). There was no difference in T-cells response against PHA in samples from healthy controls with (*N* = 1) and without combined SNPs (*N* = 5). T-cells from RA samples with both SNPs responded to MAP PPD-like with a 7.4 ± 6.7-fold increase (*N* = 5) compared to a 3.4 ± 1.8-fold increase (*N* = 5) in normal RA samples (Figure 4F). Similarly, T-cells from healthy controls with combined SNPs resulted in 1.7-fold increase (*N* = 1) when treated with MAP PPD-like compared to only 1.4 ± 0.2-fold increase (*N* = 5) in T-cells from healthy control without SNPs.

### Effect of *PTPN2:rs478582* and *PTPN22:rs2476601* on *IFN*-γ expression

**Figure 5:**
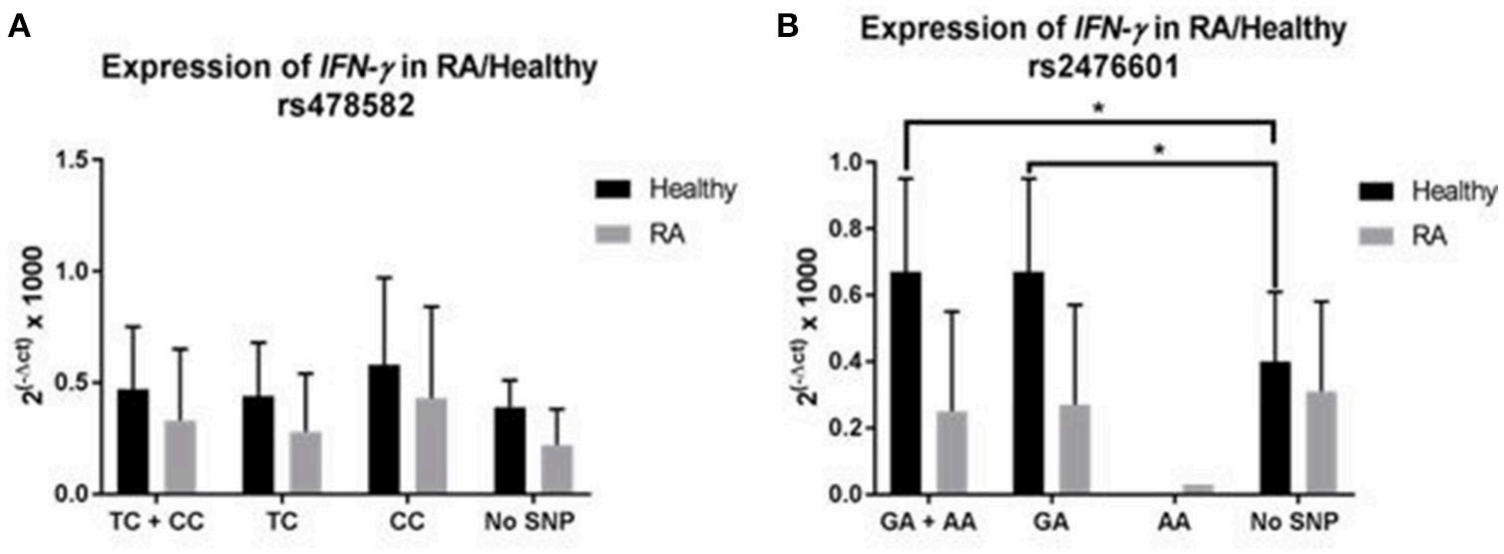
Effect of *PTPN2:rs478582*/*PTPN22:rs2476601* on *IFN-*γ Expression in RA. **(A)** *IFN-*γ expression in RA and healthy control subjects with *PTPN2:rs478582*. **(B)** *IFN-*γ expression in RA and healthy control subjects with *PTPN22:rs2476601*. ^*^P-values ≤ 0.05.

The effect of *PTPN2:rs478582* and *PTPN22:rs2476601* on *IFN-*γ expression was determined on 35 RA and 24 healthy controls (Figure 5). The average relative mRNA expression of *IFN-*γ in all samples with *PTPN2:rs478582* heterozygous (TC) or minor (CC) allele, regardless of disease, was 0.39 ± 0.31 (*N* = 38) compared to 0.28 ± 0.16 (*N* = 21) in normal (TT) samples. Specifically, the average relative mRNA expression of *IFN-*γ in all samples with *PTPN2:rs478582* minor (CC) allele was 0.48 ± 0.39 (*N* = 12). In RA samples, the average relative mRNA expression of *IFN-*γ in samples with *PTPN2:rs478582* heterozygous (TC) or minor (CC) allele was 0.33 ± 0.32 (*N* = 22), compared to 0.22 ± 0.16 in 13 normal (TT) RA samples (Figure 5A). Surprisingly, the effect of the *PTPN2:rs478582* minor (CC) allele on *IFN-*γ expression in RA samples was more significant (0.43 ± 0.41; *N* = 8). However, the average relative mRNA expression of *IFN-*γ in healthy control samples with and without *PTPN2:rs478582* heterozygous (TC) or minor (CC) allele was similar [0.47 ± 0.28 (*N* = 16), 0.39 ± 0.12 (*N* = 8), respectively (Figure 5A)]. As observed in RA samples, the effect of *PTPN2*:*rs478582* minor (CC) allele on *IFN-*γ expression in healthy controls was elevated (0.58 ± 0.39; *N* = 4). Correlation analyses were also performed to determine if *PTPN22:rs2476601* alters *IFN-*γ expression (Figure 5B). In healthy controls, the average relative mRNA expression of *IFN-*γ in samples with the heterozygous (GA) or minor allele (AA) for *PTPN22:rs2476601* was 0.67 ± 0.28 compared to 0.40 ± 0.21 in normal (GG) samples (*p*-value ≤ 0.05). There was no significant effect for *PTPN22:rs2476601* heterozygous (GA) or minor allele (AA) on *IFN-*γ expression in RA samples.

### Effect of *PTPN2:rs478582* and *PTPN22:rs2476601* on susceptibility to MAP infection

**Figure 6:**
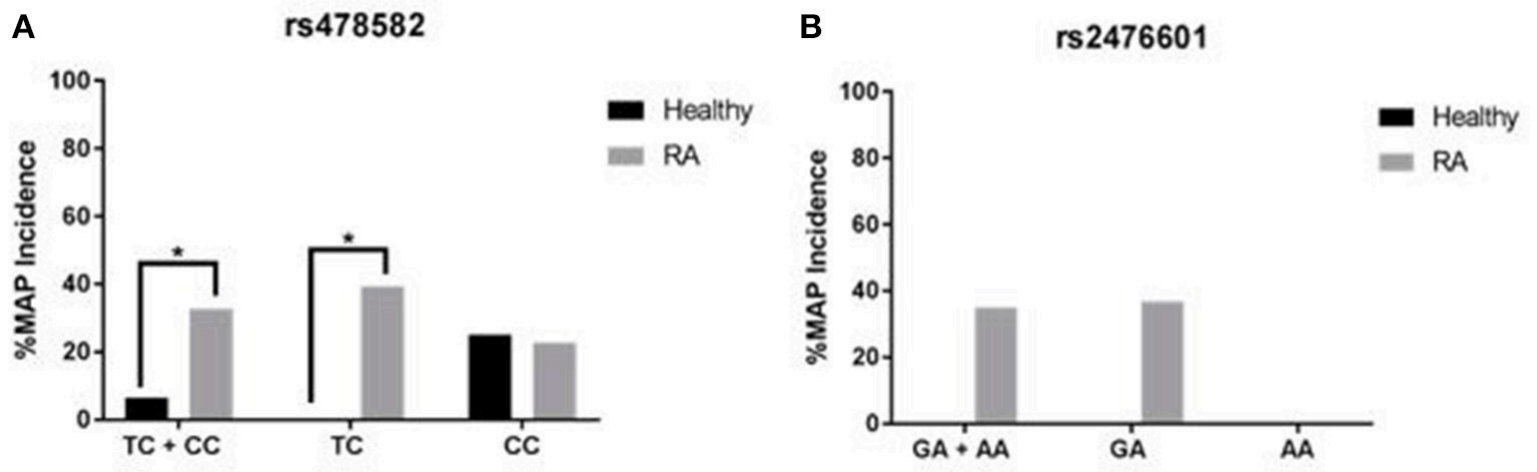
Effect of *PTPN2:rs478582* and *PTPN22:rs2476601* on Susceptibility to MAP Infection. **(A)** MAP in the blood from RA and healthy samples associated with *PTPN2:rs478582* [heterozygous allele (TC), minor allele (CC) and combined alleles (TC + CC)]. **(B)** MAP in the blood from RA and healthy samples-associated with *PTPN22:s2476601* [heterozygous allele (GA), minor allele (AA), and combined alleles (GA + AA)]. ^*^P-values ≤ 0.05.

Correlation analyses were performed to determine if *PTPN2:rs479592* in RA may affected susceptibility to MAP infection (Figure 6A). Out of 55 RA samples with either heterozygous (TC) or minor (CC) allele for *PTPN2:rs478582*, 18/55 (32.7%) were positive for *MAPbacteremia* compared to only 2/31 (6.5%; *p*-value ≤ 0.05) in healthy controls. The OR value was 7.05 (95% CI: 1.51–32.9). Specifically, MAP presence in RA samples with only heterozygous (TC) allele was 13/33 (39.3%) compared to none in 23 healthy controls samples (*p*-value ≤ 0.05).

Similarly, correlation analyses were performed to determine if *PTPN22:rs2476601* in RA may affected susceptibility to MAP infection (Figure 6B). Out of 20 RA samples with either the heterozygous or minor allele for *PTPN22:rs2476601*, 7/20 (35.0%) had *MAPbacteremia* compared to none in healthy controls. OR value of significance was 5.00 (95% CI: 0.23–106.1). Specifically, MAP presence in RA samples with heterozygous allele was 7/19 (36.8%) compared to none in healthy controls. MAP was absent in all samples with minor allele.

We also investigated the *PTPN2/22* expression in MAP positive samples. Overall, samples with *MAPbacteremia* had an average relative *PTPN2* mRNA expression of 10.0 ± 6.31 (*N* = 15) compared to 9.00 ± 6.16 (*N* = 52) in MAP-free samples, regardless of disease. In RA samples with MAP, the average relative mRNA expression of *PTPN2* was 9.53 ± 5.42 (*N* = 12) compared to 7.59 ± 5.28 (*N* = 25) in MAP-free samples. Only three healthy controls samples were positive for MAP and they had average relative mRNA expression of *PTPN2* 11.9 ± 10.57 compared to 10.3 ± 6.71 (*N* = 27) in MAP-free samples. There was no change in *PTPN22* expression in samples with or without MAP.

### Effect of combined *PTPN2:rs478582, PTPN22:rs2476601*, and MAP on *PTPN2/22* expression

The correlation of *PTPN2/22* expression in samples with either *PTPN2:rs478582* or *PTPN22:rs2476601* that had MAP presence was examined as seen in Tables 4, 5. The overall relative mRNA expression of *PTPN2* was lower in RA compared to healthy controls (8.22 ± 5.33 and 10.3 ± 6.95, respectively). The effect of *PTPN2:rs478582* on *PTPN2* gene expression in RA with heterozygous (TC) or minor (CC) allele was 8.67 ± 5.59 (*N* = 24) compared to 10.6 ± 7.47 (*N* = 23) in healthy controls with similar SNPs and lower than healthy controls without SNPs (TT) (9.49 ± 5.13; *N* = 8). In MAP positive RA samples with *PTPN2:rs478582*, the average relative mRNA expression of *PTPN2* was 9.49 ± 6.15 compared to 6.01 ± 4.70 (*N* = 8) in normal (TT) MAP-free samples. Only one healthy control sample was positive for MAP and had heterozygous (TC) allele had an average relative mRNA expression in *PTPN2* of 24.1 compared to 8.36 ± 4.42 (*N* = 4) in healthy controls without MAP presence and without the SNP.

**Table 4:** Effect of combined *PTPN2:rs478582* and MAP presence on *PTPN2* expression.

**Diagnosis**	***PTPN2*** **expression of samples with** ***PTPN2:rs478582*** **and MAP − (2**^**−ΔCT**^ **× 1,000)**				***PTPN2*** **expression of samples with** ***PTPN2:rs478582*** **and MAP + (2**^**−ΔCT**^ **× 1,000)**			
	**TT**	**TC**	**CC**	**TC + CC**	**TT**	**TC**	**CC**	**TC + CC**
RA	6.0 ± 4.7 (*N* = 8)	7.78 ± 4.67 (*N* = 10)	9.14 ± 6.84 (*N* = 7)	8.33 ± 5.5 (*N* = 17)	9.59 ± 4.89 (*N* = 5)	6.73 ± 2.57 (*N* = 5)	16.4 ± 8.17 (*N* = 2)	9.49 ± 6.15 (*N* = 7)
Healthy	10.7 ± 5.44 (*N* = 6)	10.3 ± 7.0 (*N* = 18)	11.9 ± 9.73 (*N* = 5)	10.6 ± 7.47 (*N* = 23)	5.86 ± 0.75 (*N* = 2)	NA	24.1 (*N* = 1)	24.1 (*N* = 1)

**Table 5:** Effect of combined *PTPN22:rs2476601* and MAP presence on *PTPN22* expression.

**Diagnosis**	***PTPN22*** **expression of samples with** ***PTPN22:rs2476601*** **and MAP − (2**^**−ΔCT**^ **× 1,000)**				***PTPN22*** **expression of samples with** ***PTPN22:rs478582*** **AND MAP + (2**^**−ΔCT**^ **× 1,000)**			
	**GG**	**GA**	**AA**	**GA + AA**	**GG**	**GA**	**AA**	**GA + AA**
RA	2.27 ± 1.72 (*N* = 16)	2.51 ± 1.57 (*N* = 7)	3.16 (*N* = 1)	2.59 ± 1.47 (*N* = 8)	2.8 ± 2.49 (*N* = 8)	3.12 ± 0.7 (*N* = 5)	NA	3.12 ± 0.7 (*N* = 5)
Healthy	3.22 ± 1.94 (*N* = 26)	3.4 ± 1.19 (*N* = 4)	NA	3.4 ± 1.19 (*N* = 4)	2.83 ± 1.95 (*N* = 3)	NA	NA	NA

Similarly, relative mRNA expression of *PTPN22* was also lower in RA compared to healthy controls (2.55 ± 1.74, and 3.24 ± 1.84, respectively). The average relative mRNA expression in RA with heterozygous (GA) or minor allele (AA) was 2.79 ± 1.23 (*N* = 13) compared to 3.40 ± 1.19 (*N* = 4) in healthy controls with similar SNP and normal (GG) healthy control (3.24 ± 1.91, *N* = 27). Overall, samples with MAP presence and the *PTPN22:rs2476601* heterozygous (GA) or minor (AA) allele had an average relative mRNA expression of 3.12 ± 0.70 (*N* = 5) compared to 2.85 ± 1.89 (*N* = 41) in normal MAP free samples. In MAP positive RA samples with *PTPN22:rs2476601*, the average relative mRNA expression of *PTPN22* was 3.12 ± 0.70 (*N* = 5) compared to normal MAP-free (2.24 ± 1.67; *N* = 17). None of healthy control samples has both MAP presence and *PTPN22:rs2476601*.

Only 3 RA samples had *PTPN2:rs478582, PTPN22:rs2476601* and were positive for MAP. *PTPN2/22* expression and T-cell response were not significantly different from early observation.

### Effect of combined *PTPN2:rs478582, PTPN22:rs2476601*, and MAP on *IFN-γ* expression

Overall, there was no significant difference in *IFN-*γ mRNA expression in 59 samples with or without MAP presence. The average relative mRNA expression in *IFN-*γ was 0.35 ± 0.26 in samples with MAP present, while samples without MAP presence (*N* = 45) was 0.35 ± 0.28. Similar data was observed when gene expression was evaluated in RA and healthy control samples.

**Figure 7:**
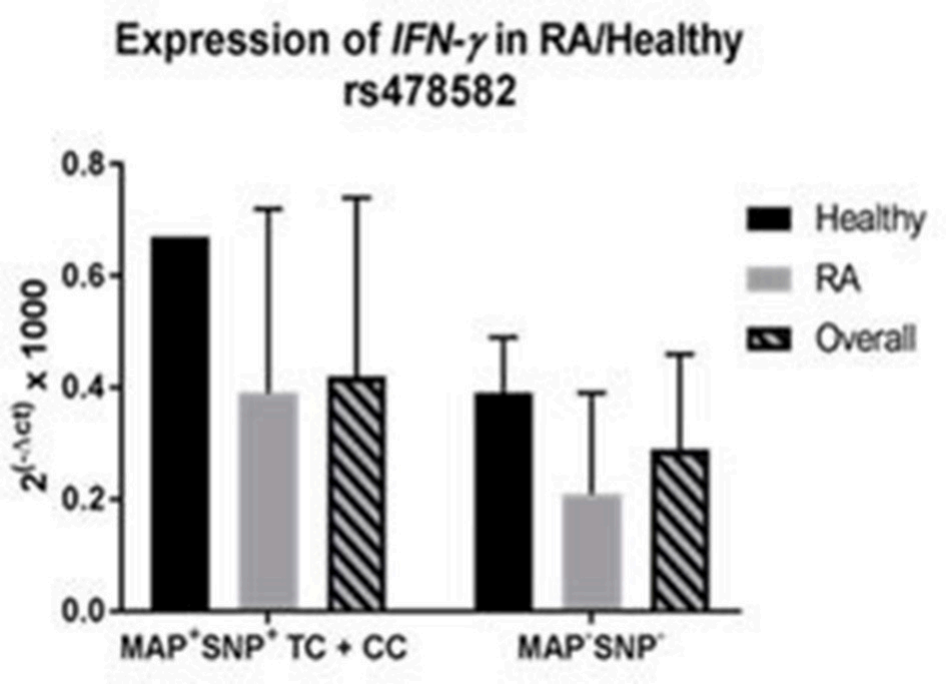
Combined Effect of MAP and *PTPN2:rs478582* on *IFN-*γ Expression in RA.

Correlation analyses were performed to determine if MAP presence with *PTPN2:rs478582* heterozygous (TC) or minor (CC) allele changes gene expression of *IFN-*γ (Figure 7). Overall, samples with MAP presence and *PTPN2:rs478582* (*N* = 7) had an average relative mRNA expression in *IFN-*γ of 0.43 ± 0.32, while samples without MAP presence and the SNP (*N* = 14) was 0.29 ± 0.17. In RA samples, the average relative mRNA expression in *IFN-*γ in samples with MAP presence and *PTPN2:rs478582* (*N* = 6) was 0.39 ± 0.33 compared to 0.21 ±0.18 (*N* = 8) in RA samples without MAP presence and without the SNP. In healthy controls, the average relative mRNA expression in *IFN-*γ in samples with MAP presence and *PTPN2:rs478582* (*N* = 1) was 0.67 compared to 0.39 ± 0.10 in healthy controls without MAP presence and without the SNP (*N* = 6).

Correlation analyses were also performed to determine if MAP presence with *PTPN22:rs2476601* heterozygous (GA) or minor (AA) allele changes gene expression of *IFN-*γ. Overall, samples with MAP presence and *PTPN22:rs2476601* (*N* = 5) had an average relative mRNA expression in *IFN-*γ of 0.15 ± 0.07, while samples without MAP presence and the SNP (*N* = 34) was 0.32 ± 0.22. In healthy controls, there were no samples with both MAP presence and *PTPN22:rs2476601* together. In RA patients, the average relative mRNA expression in *IFN-*γ in samples with MAP presence and the *PTPN22:rs2476601* SNP (*N* = 5) was 0.15 ± 0.07 compared to 0.27 ± 0.24 in RA patients without MAP presence and without the SNP (*N* = 17).

Only 3 RA samples had *PTPN2:rs478582, PTPN22:rs2476601* and were positive for MAP. *IFN-*γ expression and T-cell response were not significantly different from early observation.

### Effect of medication to susceptibility to *MAPbacteremia*

The effect of the medications taken by RA participants, as shown in Table 1, were evaluated for the susceptibility of MAP. Four different medication groups were studied for MAP susceptibility: hydroxychloroquine (TLR repressor), methotrexate (anti-folate), prednisone (steroid), and anti-TNF-α medications (Humira®, Enbrel®, Simponi®, and Cimzia®). Out of 14 RA patients on hydroxychloroquine, 6 (42.9%) were positive for *MAPbacteremia*, while 12 out of 37 (32.4%) of RA patients on methotrexate also were positive for *MAPbacteremia*. For RA patients on prednisone, 4 out of 16 (25.0%) of RA patients were positive for *MAPbacteremia*, while 6 out of 23 (26.1%) RA patients on anti-TNF-α medications (Humira®, Enbrel®, Simponi®, and Cimzia®) also were positive for *MAPbacteremia*.

## Discussion

Extensive efforts are ongoing to investigate pathogenesis and effective treatment for inflammatory diseases. Current medications are expensive and the side effects are lengthy. For example, RA etiology remains unknown, but there are established protocols for diagnosis of and management of symptoms. However, the side effects of all RA medications are serious and undesirable (Majithia and Geraci, 2007; Dixon et al., 2010; Smolen et al., 2016). Therefore, it is imperative that the pathogenesis of RA is deciphered in order to develop protocols for accurate and early detection and treatment of the disease. RA patients suffer from elevation of pro-inflammatory cytokines such as IFN-γ and TNF-α and their impact on apoptosis and development of chronic inflammation (Majithia and Geraci, 2007; McInnes and Schett, 2011; Smolen et al., 2016). Only environmental factors and genetic predisposition mutations have been linked to RA (Klareskog et al., 2011; Yarwood et al., 2014; Fisher et al., 2015; Smolen et al., 2016). This study is focused on investigating the effect of SNPs on key negative regulators genes such as *PTPN2/22* expression and their impact on upregulation of pro-inflammatory cytokines, apoptosis and inflammation. We hypothesized that heterozygous and/or homozygous minor allele mutation(s) in health-keeping genes such as *PTPN2/22* in RA lead to elevated IFN-γ, TNF-α, apoptosis, and development of inflammation. To our knowledge, this is the first study designed to elucidate the molecular cause of inflammation in RA in association with environmental triggers such as MAP. The latter has been associated with similar inflammation in CD, T1D, multiple sclerosis, and others (Naser et al., 2009a,b, 2013; Masala et al., 2011; Sharp et al., 2015). This study is first to report the detection of MAP DNA in more than of one third of RA patients (Figure 2B). The data is significant, intriguing, and should be a motive to expand future investigations to include a larger pool of samples. As well-advertised, the incidence of *M. tuberculosis* infection in RA, is among the most accepted side effect of the treatment (Dixon et al., 2010; McInnes and Schett, 2011; Smolen et al., 2016). Therefore, detection of MAP infection in RA patients should be investigated further as to whether it is a complication of the treatment or a possible culprit of the disease. Although MAP *IS900* DNA is good in detecting MAP presence in patient samples, it does not provide information about the MAP bacteria viability. This in turn does not show accurate status of either active or previous infection in the patient sample. Thus, culturing of the blood from RA patients is necessary to determine if an active MAP infection is present in the patients.

Genetic predisposing is key for susceptibility to disease, severity of inflammation and possible ability to respond to treatment. Due to the large number of published SNPs in *PTPN2/22*, we selected 9 SNPs in this study based on shared occurrence in other diseases with similar approved treatment protocol (McInnes and Schett, 2011; Yarwood et al., 2014; Sharp et al., 2015; Smolen et al., 2016). Specifically, we focused on SNPs in *PTPN2/22*, which increase susceptibility to RA and CD. The latter is well-investigated in our laboratory in association with MAP (Naser et al., 2004, 2009b; Sharp et al., 2015). This study identified *PTPN2:rs478582* to be significant in RA (*p*-values ≤ 0.05, OR = 2.28) compared to healthy controls (Figure 3A). Similarly, *PTPN22:rs2476601* was significant in RA (*p*-values ≤ 0.05, OR = 5.90) compared to healthy controls (Figure 3B). The data specifically linked *PTPN2:rs478582* minor (CC) allele to be more significantly associated with RA (*p*-values ≤ 0.05). In short, our data suggest to clinicians that minor (CC) allele in *PTPN2* increases the risk of acquiring RA by a fold of 2.1. Moreover, *PTPN22:rs2476601* heterozygous (GA) allele in *PTPN22* was more significant in RA (*p*-values ≤ 0.05), indicating that patients with this SNP are at risk of acquiring RA by a fold of 4.3. Further examination of RA genotyping showed that patient samples with both *PTPN2:rs478582* and *PTPN22:rs2476601* alleles (regardless of heterozygous or minor alleles) showed to be more significant (*p*-values ≤ 0.05) compared to healthy controls, showing a 6.5-fold increased risk of developing RA. Some of the limitations of looking into SNPs in a diverse population, such as from this study, is that it is difficult to determine the alterations of allelic distribution between different population groups. Thus, it is important that further population studies that focus on examining *PTPN2/22* SNPs from other subpopulation groups, such as race, country of origin, and age/gender in RA patients should be done.

The effect of SNPs on *PTPN2/22* gene expression and function have been debated heavily in the literature (Vang et al., 2005; Serrano et al., 2013; Spalinger et al., 2013; Sharp et al., 2015). This study demonstrated that RA samples with either *PTPN2:rs478582*/*PTPN22:rs2476601* heterozygous or minor alleles could potentially alter the *PTPN2/22* gene or the protein activity of PTPN2/22 in T-cells, thus could possibly void the negative regulatory function of PTPN2/22. The effects of *PTPN2:rs478582*/*PTPN22:rs2476601* in *PTPN2/22* were also examined further to determine the effect on T-cell and production of IFN-γ.

Since *PTPN2/22* is found in all T-cell types, we decided not to segregate the T-cell subpopulations and instead look into total T-cell activity. However, further studies need to be done on the effects of SNPs in *PTPN2/22* in different subpopulations of T-cells. Stimulation of T-cells from RA samples associated with *PTPN2:rs478582* and induced with PHA led marked increase in T-cell proliferation. T-cells from RA patients associated *PTPN22:rs2476601* had even more significant increase in T-cell proliferation (*p*-values ≤ 0.05). Moreover, it was shockingly surprising to see T-cell reactivity response treatment with MAP PPD-like. Specifically, T-cells, isolated from the blood of RA patients associated with *PTPN2:rs478582*/*PTPN22:rs2476601* combined SNPs, proliferated by several folds more than those cells from health controls. Thus, T-cells from RA samples associated with SNPs in *PTPN2/22* seem to demonstrate three characteristics: first, they are hyperactive, second they seem to lack a negative feedback control, and third they reacted to MAP PPD-like significantly indicating prior exposure to MAP antigens. Hyperactive T-cells with lack of negative feedback control may explain the marked increase in pro-inflammatory cytokines such as TNF-α and IFN-γ in RA. The examination of T-cells in this study has been an exploratory study, thus it is necessary to examine bigger populations of RA and health control subjects in the future. Along with this, further investigation on the outcome of the other immune cells, such as B-cells, NK cells, and macrophages, need to be examined in RA patients with SNPs in *PTPN2/22* to conclude how the hyper-proliferative T-cells react to other immune cells.

The hyperactivity to MAP PPD-like in the RA T-cells may be correlated to presence of MAP in RA samples and possibly activation of *M. tuberculosis* in some RA patients with biologic drugs treatments. The study provided more evidence that SNPs in *PTPN2/22* may have increased susceptibility to MAP infection as shown in Figure 6. Specifically, *PTPN2:rs478582* correlated with MAP infection in 32.7% (OR = 7.05) RA patients. Similarly, *PTPN22:rs2476601* correlated with MAP infection in 35.0% (OR = 5.00) RA patients (*p*-values ≤ 0.05). The data also demonstrated that presence of MAP did not alter *PTPN2/22* expression.

To elucidate whether medications may have any effect on the outcome of this study, we examined the effect of current medications taken by the RA participants on the risk of susceptibility to MAP infection. As shown in Table 1, out of all the DMARDs (hydroxychloroquine, methotrexate, prednisone, Humira®, Enbrel®, etc.) that the RA patients were on, hydroxychloroquine was found to increase MAP susceptibility the most in RA patients by a 1.3-fold change compared to RA patients not on hydroxychloroquine and had *MAPbacteremia*. These findings suggest that more investigation is needed by testing larger number of patients with RA. We also discovered that *IFN-*γ expression was lower in RA patients who are on DMARDs treatment compared to RA patients who are on different treatments (Table 1). Specifically, blood samples from RA patients treated with Humira® expressed lower *IFN-*γ (0.15 ± 0.10, *N* = 11) compared to blood samples from other RA patients (0.29 ± 0.27; *p*-values ≤ 0.05) or even healthy controls (0.44 ± 0.24; *p*-values ≤ 0.05). Moreover, RA samples associated with *PTPN2:rs478582*/*PTPN22:rs2476601* heterozygous or minor alleles had higher *IFN-*γ expression than RA group without (Figure 5). These finding demonstrates that SNPs in *PTPN2/22* may led to elevated IFN-γ levels and inflammation in RA patients. However, since we focused only on *PTPN2/22* on the control of *IFN-*γ expression in this study, we did not examine the other cytokines that control IFN-γ production. Further investigation is needed to examine the effects of both pro-inflammatory cytokines, such as TNF-α, and anti-inflammatory cytokines, such as IL-6, in subjects with SNPs in *PTPN2/22*.

Overall, the data supports our hypothesis that SNPs in *PTPN2/22* leads to loss functions of these genes resulting in hyper-proliferative T-cells and increase susceptibility to *Mycobacteria* including MAP. Along with genetic testing for SNPs and proper treatment, personalized treatment for RA is plausible. More studies are encouraged to explore the incidence and impact of these SNPs on health keeping genes and susceptibility to infection in RA.

## Author contributions

RS, Ph.D. candidate, has performed all experiments, and collected and analyzed all data in this study. He was also instrumental in designing the experiments and writing the manuscript. SB is the clinical coordinator in this study and has supervised recruitment of subjects, collection of clinical samples, and transmitting of relevant data to the investigators. She played a vital role in analyzing the data and revising the manuscript. SN is the corresponding author on the manuscript and the primary investigator of the study. He managed the entire study, supervised all experiments, and helped analyze and interpret data and writing of the manuscript.

### Conflict of interest statement

The authors declare that the research was conducted in the absence of any commercial or financial relationships that could be construed as a potential conflict of interest.
